# Targeting MKK3 as a novel anticancer strategy: molecular mechanisms and therapeutical implications

**DOI:** 10.1038/cddis.2014.591

**Published:** 2015-01-29

**Authors:** S Baldari, V Ubertini, A Garufi, G D'Orazi, G Bossi

**Affiliations:** 1 Experimental Oncology Laboratories, Regina Elena National Cancer Institute, Rome, Italy; 2 Department of Medical, Oral and Biotechnological Sciences, University “G. d'Annunzio”, Chieti, Italy; 3 Laboratory of Medical Physics and Expert Systems, Regina Elena National Cancer Institute, Rome, Italy

## Abstract

Mitogen-activated protein kinase kinase 3 (MAP2K3, MKK3) is a member of the dual specificity protein kinase group that belongs to the MAP kinase kinase family. This kinase is activated by mitogenic or stress-inducing stimuli and participates in the MAP kinase-mediated signaling cascade, leading to cell proliferation and survival. Several studies highlighted a critical role for MKK3 in tumor progression and invasion, and we previously identified MKK3 as transcriptional target of mutant (mut) p53 to sustain cell proliferation and survival, thus rendering MKK3 a promising target for anticancer therapies. Here, we found that targeting MKK3 with RNA interference, in both wild-type (wt) and mutp53-carrying cells, induced endoplasmic reticulum stress and autophagy that, respectively, contributed to stabilize wtp53 and degrade mutp53. MKK3 depletion reduced cancer cell proliferation and viability, whereas no significant effects were observed in normal cellular context. Noteworthy, MKK3 depletion in combination with chemotherapeutic agents increased tumor cell response to the drugs, in both wtp53 and mutp53 cancer cells, as demonstrated by enhanced poly (ADP-ribose) polymerase cleavage and reduced clonogenic ability *in vitro*. In addition, MKK3 depletion reduced tumor growth and improved biological response to chemotherapeutic *in vivo*. The overall results indicate MKK3 as a novel promising molecular target for the development of more efficient anticancer treatments in both wtp53- and mutp53-carrying tumors.

MKK3 is a dual specificity protein kinase that belongs to the mitogen-activated protein kinase (MAPK) signaling pathway, an important signal transduction system that participates in a plethora of cellular programs, including cell differentiation, movement, division, and death.^1^ In particular, MKK3 is activated upon different forms of stressful stimuli and inflammatory cytokines^2, 3^ through phosphorylation of serine and threonine residues at sites Ser189 and Thr193^4^ by several upstream MAPK kinases, such as mixed lineage kinases, transforming growth factor-b-activated kinase 1, and apoptosis signal-regulating kinase 1.^5^ Once activated, MKK3 specifically phosphorylates and activates p38MAPK at its activation site Thr-Gly-Tyr.^2, 3, 4, 6, 7^

Recent findings revealed that MKK3 has relevant role in tumor invasion and progression of gliomas and breast tumors.^8^ Accordingly, we previously demonstrated that MKK3 is a novel upregulated target gene of mutant (mut)-p53 gain-of-function activity, and that MKK3 knockdown strongly reduces cell proliferation and survival of mutp53-bearing and p53-null human tumor cells.^9^ Interestingly, other studies demonstrated that MAPK14/p38MAPK is required for cell proliferation and survival, and that its inhibition leads to cell cycle arrest and autophagy-mediated cell death.^10^ Autophagy is an efficient degradation process that occurs at a basal rate in most cells, in which it helps to maintain homeostasis, acting as a cytoplasmic quality-control mechanism able to eliminate unnecessary, aggregating-prone proteins and injured organelles.^11, 12, 13, 14, 15^ Autophagy is also responsible for the survival response to growth-limiting conditions, such as nutrient deprivation. Under these stressful conditions, autophagy enhances its function as a survival mechanism, by which cellular components are sequestered into a double-membrane vesicle, delivered to the lysosome system for final digestion^16, 17, 18, 19^ and released for recycling of nutrients necessary to maintain protein synthesis, to produce substrates for oxidation and for ATP synthesis in the mitochondria^20^ and to contribute to the inhibition of apoptosis.^21^ However, non-productive, uncontrolled, or prolonged autophagy leads to what has been designated 'autophagic cell death'.^22, 23^ Several metabolic stresses can induce autophagy, such as hypoxia, oxidative stress, expression of aggregate-prone proteins, and glucose deprivation.^24^ An increasing number of studies also suggest that autophagy could be induced in consequence of the unfolded protein response, which is the major endoplasmic reticulum (ER) stress pathway.^25^ Indeed, ER stress stimulates autophagy through the PKR-like ER kinase (PERK)/eukaryotic translation initiation factor 2*α* (eIF2*α*) and Inositol-requiring Enzyme 1 (IRE1)/ c-Jun N-terminal kinase 1 pathways. PERK/eIF2*α* phosphorylation is essential for the transcription of key autophagy-associated genes during ER stress and may mediate the polyglutamine-induced microtubule-associated protein 1 light chain 3 (LC3) conversion, which is a marker of autophagy.^26^

Our previous studies suggested MKK3 as a general molecular player required to sustain cell proliferation and survival not only in mutp53-bearing but also in p53-null cancer lines.^9^ Here, we wanted to evaluate whether MKK3 played a role also in wild-type (wt) p53-bearing cells and its impact on both mup53 and wtp53 tumor cell response to anticancer drugs, investigating the molecular mechanisms involved in the biological outcomes upon MKK3 depletion. We found that MKK3 depletion reduced cell proliferation and survival of wtp53 cancer cells without affecting normal untransformed cells. Indeed, MKK3 depletion induced ER stress that correlated with stabilization and activation of wtp53. Moreover, MKK3 depletion induced cell autophagy that contributed to the degradation of mutp53, in agreement with recent studies.^24, 27^ Furthermore, at biological level, MKK3 depletion in combination with chemotherapy reduces clonogenicity in both wtp53 and mutp53 cancer cells and induced higher *in vivo* anti-tumoral effects in a xenograft tumor model, when compared with drug treatment alone. The overall results revealed that, in the adopted *in vitro* and *in vivo* experimental tumor models, the MKK3 targeting might constitute an interesting strategy to improve anticancer treatment in both wtp53 and mutp53 cancer cells.

## Results

### MKK3 depletion reduces cell proliferation and viability in wtp53-bearing cancer but not normal cells

**Figure 1:**
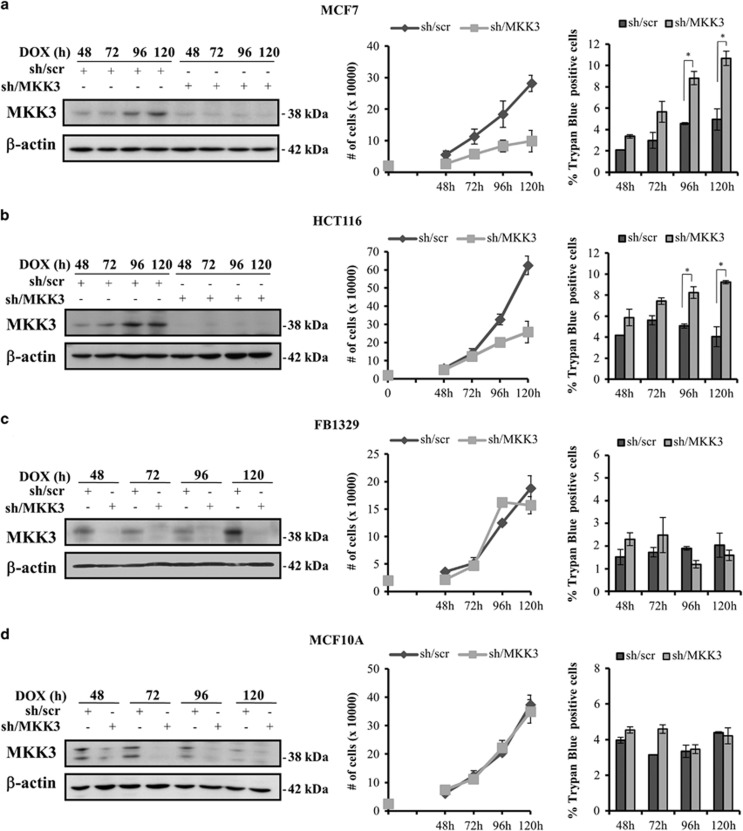
MKK3 depletion is detrimental to cancer but not normal cell proliferation and survival. Efficient MKK3 depletion was achieved in all tested lines (**a**–**d**, left panels): Engineered MCF7 (**a**), HCT116 (**b**), FB1329 (**c**) MCF10A (**d**) -sh/scr and -sh/MKK3 sublines (1.5 × 10^5^ cells/60 mm dish) were challenged with DOX (1.0 *μ*g/ml) and cells collected time dependently (48, 72, 96 and 120 h) to assess the MKK3 depletion efficiency. Then, protein lysates (30 *μ*g /lane) were resolved in SDS–polyacrylamide gel electrophoresis and filter analyzed by western blot analysis with specific anti-MKK3 and anti-*β*-actin (loading control) antibodies. MKK3 depletion affects cell proliferation and viability of wtp53 tumor but not normal cells (**a–d**, *right panels*). Engineered MCF7 (**a**), HCT116 (**b**), FB1329 (**c**) MCF10A (**d**) -sh/scr and -sh/MKK3 sublines were seeded (2 × 10^4^ cells/6-well plates) in DOX conditions. At the indicated time points, cells were harvested and quantified for viable and death cells by Trypan blue exclusion assays. Results are reported as means and S.D. of three independent experiments. *The significance (*P*<0.05) of death percent in sh/MKK3 with respect to sh/scr subline

We previously showed that MKK3 is a general required factor to sustain cell proliferation and survival in mut- and null-p53 human cancer cell lines.^9^ Here, we aimed to explore whether MKK3 could have similar roles in wtp53 cell-context with a panel of human cancer (MFC7, HCT116) and primary non-transformed (FB1329, MCF10A) cell lines. All cell lines have engineered with conditional tetracycline (TET)-OFF lentiviral-based system carrying shRNA sequences specific to MKK3 (sh/MKK3) or RNA interference control (short hairpin/scramble (sh/scr)), and MKK3 depletion was obtained after treatment with TET analogous doxycycline (DOX), as previously described.^9^ We first studied the biological effects upon MKK3 depletion, in a time-dependent manner. Efficient MKK3 depletion (sh/MKK3) was achieved as early as 48 h upon DOX delivery in all tested cell lines, with respect to control cells (sh/scr), and maintained throughout time (Figures 1a–d, *left panels*). MKK3 depletion reduced cell proliferation and significantly increased cell death in both MCF7 and HCT116 cells (Figures 1a and b, middle and right panels). Interestingly, MKK3 depletion did not modify proliferation and viability of primary foreskin fibroblasts (FB1329) and normal mammary epithelial (MCF10A) cells (Figures 1c and d, middle and right panels).

Results suggest that MKK3 depletion exclusively hampers cell proliferation and viability of cancer but not normal untransformed cells.

### MKK3 depletion stabilizes wtp53 protein

**Figure 2:**
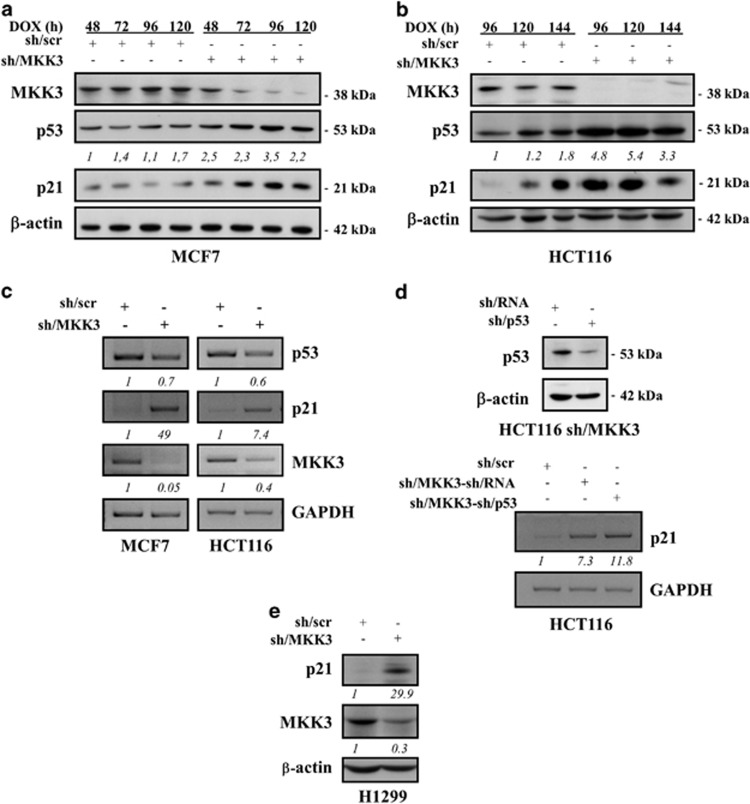
MKK3 depletion stabilizes wtp53 protein. Engineered MCF7 (**a**) and HCT116 (**b**) -sh/scr and -sh/MKK3 sublines were cultured with DOX (1.0 μg/ml) and collected at the indicated time points. Protein lysates (30 μg/lane) were analyzed by western blot analysis with anti-MKK3-, anti-p53-, anti-p21-, and anti-*β*-actin (loading control)-specific antibodies. Densitometry was performed with ImageJ software and relative p53 band intensity normalized to *β*-actin and quantified with respect to controls (sh-scr) set to 1.0. (**c**) Semi-quantitative RT-PCR was performed on total RNAs isolated from engineered MCF7 (left panel) and HCT116 (right panel) -sh/scr and sh/MKK3 sublines maintained 72 h with DOX. PCR was performed with specific set of primers. GAPDH was used as housekeeping gene. RT-PCR images were acquired by Bio-Rad Universal Hood II gel-imager. Densitometry was performed with ImageJ software and relative p53, p21, and MKK3 band intensity normalized to GAPDH and quantified with respect to controls (sh-scr) set to 1.0. (**d**) Upper panel: HCT116-sh/MKK3 sublines were transfected with sh/p53 or control sh/RNA carrying vector and efficiency of p53 depletion detected by western blotting (30 *μ*g/lane). Lower panel: HCT116-sh/scr, -sh/MKK3-sh/RNA, and -sh/MKK3-sh/p53 were cultured 72 h with DOX, then total RNAs were isolated and semi-quantitative RT-PCR performed with set of primers specific to p21 and GAPDH (housekeeping gene). Images were acquired by Bio-Rad Universal Hood II gel-imager, and densitometry performed with ImageJ software. Relative p21 band intensity was normalized to GAPDH and quantified with respect to controls (sh-scr) set to 1.0. (**e**) Engineered H1299-sh/scr and -sh/MKK3 sublines were cultured with DOX (1.0 *μ*g/ml) and collected 96 h later. Protein lysates (30 *μ*g/lane) were analyzed by western blot analysis with anti-MKK3-, anti-p21- and anti-*β*-actin (loading control)-specific antibodies. Densitometry was performed with ImageJ software and relative p21 and MKK3 band intensity normalized to β-actin and quantified with respect to controls (sh-scr) set to 1.0

We next wanted to examine which molecular mechanisms could be involved in the biological outcome of MKK3 depletion. To this aim, we first analyzed whether MKK3 depletion could impact on wtp53 activity. We found that MKK3 depletion raised wtp53 protein levels in MCF7 and HCT116 cells (Figures 2a and b). The effect on wtp53 stabilization was likely at protein levels because p53 mRNA was not modified by MKK3 interference (Figure 2c). Of note, wtp53 stabilization upon MKK3 depletion correlated with induced expression of p21 at both protein (Figures 2a and b) and mRNA (Figure 2c) levels. To investigate whether wtp53 is involved in the p21-induced gene expression, further assays were performed in double sh/MKK3 and sh/p53 knockdown cells. Figure 2d shows that significant p21 expression occurred in p53-depleted cells, thus indicating that p53 is not responsible of p21 induction in MKK3 knockdown cells. This observation was further confirmed by similar experiments performed in H1299 p53-null cancer cells (Figure 2e).

### MKK3 depletion induces autophagy and ER stress in wtp53 cancer cells

**Figure 3:**
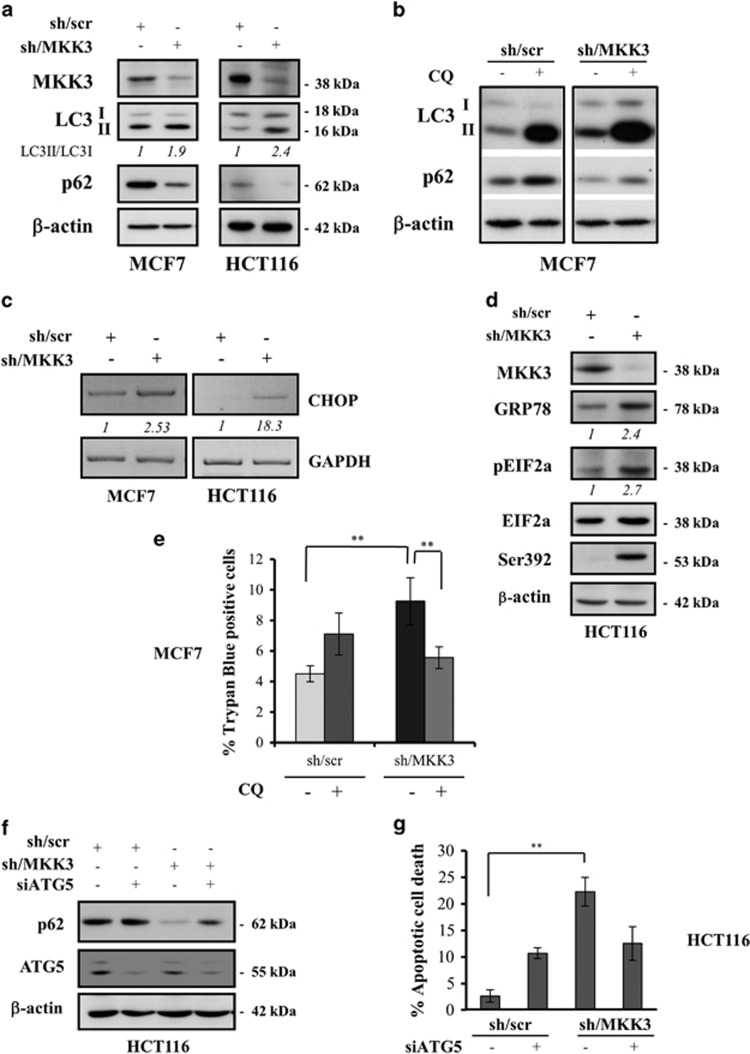
MKK3 depletion induces autophagic cell death and ER stress in wtp53 cancer cells. Markers of autophagy LC3-I, LC3-II, and p62 upon MKK3 depletion were assessed by western blotting (**a**) in wtp53 MCF7 (*l*eft panel) and HCT116 (right panel) engineered -sh/scr and sh/MKK3 sublines. Seeded cells (1.5 × 10^5^ cells/60 mm dish) were collected at 72 h post DOX delivery, and protein lysates (15 *μ*g /lane) resolved in SDS–polyacrylamide gel electrophoresis (PAGE) and probed with anti-MKK3-, anti-LC3-, and anti-SQSTM1/p62-specific antibodies. Densitometry analyses were performed with ImageJ software and LC3-II band intensity normalized to *β*-actin and quantified with respect to control tumors (sh/scr) set to 1.0. (**b**) MCF7-sh/scr and -sh/MKK3 sublines cultured in DOX condition (72 h) in the presence/absence of 25 *μ*M CQ (48 h), then protein lysate were analyzed by western blot analysis with antibodies specific to LC3, SQSTM1/p62, and *β* actin (loading control). (**c**) Engineered sh/MKK3 and sh/scr MCF7 and HCT116 cultured 48 h with DOX were collected and total RNAs analyzed by RT-PCR with set of primers specific to CHOP and GAPDH (housekeeping gene). Densitometry was performed with the ImageJ software and relative CHOP mRNA levels were normalized to GAPDH and quantified with respect to control tumors (sh-scr) set to 1.0. (**d**) Engineered sh/scr and sh/MKK3 HCT116 cell lines were maintained in DOX condition for 36 h, then cells were collected and protein lysates (30 μg/lane) analyzed by western blot for the presence of ER stress proteins: phosphorylated EIF2A protein was evaluated using phospho-specific antibodies. Total amount of EIF2A was determined using anti-EIF2A antibody. GRP78/Bip was also used as marker of ER stress. Ser392 was analyzed as p53 stabilization marker. *β*-Actin was used as loading control. Densitometry was performed with the ImageJ software and relative ER stress marker protein levels were normalized to actin and quantified with respect to control tumors (sh-scr) set to 1.0. (**e**) Autophagy inhibition rescues the cell death induced by MKK3 depletion. Engineered MCF7-sh/scr and -sh/MKK3 sublines were seeded (1.5 × 10^5^ cells/60 mm dish) in DOX condition for 24 h then treated/untreated with CQ (25 *μ*M) and collected after 96 h of total DOX induction. Cell viability was evaluated with trypan blue exclusion assay. Results are reported as mean±S.D. of three independent experiments. Significance was assessed by Student's *t*-test, **P<0.01. (**f**) Engineered HCT116-sh/scr and -sh/MKK3 sublines were seeded (1.5 × 10^5^ cells/60 mm dish) and maintained in DOX condition for 48 h, then transfected with siRNA for ATG5 (si-ATG5) or with control siRNA (si-ctr). Forty-eight hours after transfection, cells were collected and protein lysates (15 μg/lane) resolved in SDS-PAGE and probed with anti-ATG5- and anti-SQSTM1/p62-specific antibodies. *β*-Actin was used as loading control. (**g**) Engineered HCT116-sh/scr and -sh/MKK3 sublines, treated as in **f**, were collected after 96 h of DOX induction and then stained with DAPI and ethidium bromide and analyzed by fluorescent microscope. The number of apoptotic dead cells was calculated and reported as a percentage of the total number of cell counted. Results are reported as means and S.D. of three independent experiments. **The significance (*P*<0.01) of death percent in sh/MKK3 with respect to sh/scr subline

Microscopical examination revealed that MKK3 depletion induces the formation of large cytoplasmic vacuoles in cancer but not normal cells (data not shown). Because autophagic degeneration is always accompanied by cytoplasmic vacuolization,^28^ we asked whether MKK3 silencing might induce autophagy. To evaluate this mechanism, we analyzed microtubule-associated protein 1 LC3 conversion by western immunoblotting. Increased LC3 levels were observed in MCF7 and HCT116 cells upon MKK3 depletion, as evidenced by densitometric analyses of LC3-II/I ratio (Figure 3a). As autophagy is a dynamic process that begins with autophagosomes generation and terminates with their degradation in lysosomes, we investigated the autophagic flux by detecting the expression level of SQSTM1/p62, a clear marker of autophagy being itself incorporated into the completed autophagosome and then degraded in autolysosomes.^29^ Marked p62 degradation was observed along with increased LC3-II levels after MKK3 depletion, indicating the existence of the autophagic flux (Figure 3a).

To further assess the autophagic flux in MKK3-depleted cells, confirmatory assays were performed with chloroquine (CQ), an inhibitor of the final stages of autophagy.^30^ As shown in Figure 3b, CQ induces higher accumulation of LC3-II and rescues p62 degradation in sh/MKK3 cells with respect to sh/scr-treated cells.

An increasing number of studies suggest that autophagy could be induced in consequence of the unfolded protein response, which is the major endoplasmic reticulum (ER) stress pathway.^25^ Here, we found that the mRNA level of CCAAT-enhancer-binding protein homologous protein (CHOP), a clear marker of ER stress^31^ was greatly increased after MKK3 depletion in both MCF7 and HCT116 cells (Figure 3c). In agreement with ER stress induction, MKK3 depletion increased the levels of glucose-regulated protein 78/immunoglobulin heavy chain-binding protein (GRP78/Bip) protein and induced eIF2*α* phosphorylation (Figure 3d). The activation of ER stress pathway after MKK3 depletion correlated with wtp53 activation as confirmed by p53 phosphorylation at Ser392 (Figure 3d). Furthermore, we tested the impact of autophagy on cell viability by using CQ. As shown in Figure 3e, the increased cell death upon MKK3 depletion was significantly counteracted by blocking autophagy with CQ.

To further confirm whether MKK3 depletion induces autophagic cell death, small RNA interference approaches were adopted to knockdown the essential autophagic gene *ATG5*, a member of the ATG family necessary for autophagosome elongation.^32^ Efficient autophagy protein 5 (ATG5) silencing rescues p62 degradation, as readout of autophagic process inhibition (Figure 3f), and significantly reduces the apoptotic cell death in MKK3-depleted cells (Figure 3g), morphologically distinguished by severe chromatin condensation and nuclear fragmentation upon ethidium bromide cellular uptake, a marker of plasma membrane disruption.

Results suggest that MKK3 depletion triggers ER stress pathway and autophagic cell death in wtp53 cancer cells.

### MKK3 depletion reduces mutp53 protein levels through autophagy

**Figure 4:**
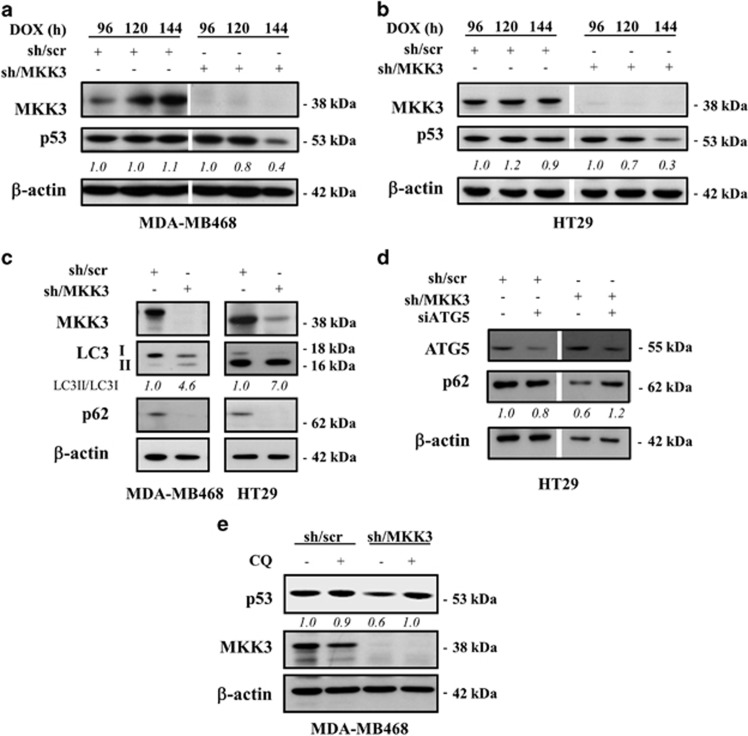
MKK3 depletion reduces mutp53 protein levels through autophagy. Engineered MDA-MB468 (**a**) and HT29 (**b**) -sh/scr and -sh/MKK3 sublines were cultured with DOX (1.0 μg/ml) and collected at indicated time points. Protein lysates (30 μg/lane) were analyzed by western blot analysis with anti-MKK3-, anti-p53-, and anti-*β*-actin (loading control)-specific antibodies. Densitometry was performed with ImageJ software and relative p53 band intensity normalized to *β*-actin and quantified with respect to controls (sh-scr) set to 1.0. (**c**) Markers of autophagy LC3-I, LC3-II, and p62 upon MKK3 depletion were assessed by western blotting in mutp53 MDA-MB468 (left panel) and HT29 (right panel) engineered -sh/scr and sh/MKK3 sublines. Seeded cells (1.5 × 10^5^ cells/60 mm dish) were collected at 120 h post DOX delivery, and protein lysates (15 μg /lane) resolved in SDS–polyacrylamide gel electrophoresis (PAGE) and probed with anti-MKK3-, anti-LC3-, and anti-SQSTM1/p62-specific antibodies. Densitometry analyses were performed with ImageJ software and LC3-II band intensity normalized to *β*-actin and quantified with respect to control tumors (sh/scr) set to 1.0. (**d**) Engineered HT29-sh/scr and -sh/MKK3 sublines were seeded (1.5 × 10^5^ cells/60 mm dish) and maintained in DOX condition for 72 h, then transfected with siRNA for ATG5 (si-ATG5) or with control siRNA (si-ctr). Forty-eight hours after transfection, cells were collected and protein lysates (15 μg /lane) resolved in SDS-PAGE and probed with anti-ATG5- and anti-SQSTM1/p62-specific antibodies. *β*-Actin was used as loading control. Densitometry analyses were performed with ImageJ software and anti-SQSTM1/p62 band intensity normalized to β-actin and quantified with respect to control tumors (sh/scr) set to 1.0. (**e**) MDA-MB468 sh/scr and sh/MKK3 sublines were cultured 120 h with DOX and, in the last 48 h, treated/untreated with CQ (25 μM). Afterwards, cells were collected and lysate analyzed by western blot analysis with anti-p53-, anti-MKK3-, and anti-β actin (loading control)-specific antibodies

Recent studies disclosed the role of autophagy in mutp53 degradation^24, 27^ and we above found that MKK3 depletion induced autophagy in wtp53 cells. Therefore, we next aimed to test whether MKK3 knockdown could influence mutp53 levels and autophagy. We found that mutp53 protein levels underwent efficient reduction upon MKK3 depletion in both MDA-MB468 and HT29 cells (Figures 4a and b). Moreover, MKK3 depletion induced autophagy also in mutp53 cells, as well as above for wtp53 cells, as evidenced by LC3-II induction and p62 degradation (Figure 4c). Noteworthy, a significant delay in autophagy induction was observed in mutp53 cells upon MKK3 depletion when compared with wtp53 cells (120–144 h *versus* 72–96 h, respectively), which correlates with mutp53 protein reduction. Genetic approaches showed that ATG5 depletion rescues the p62 degradation in mutp53 sh/MKK3 cells further confirming autophagy (Figure 4d). Moreover, importantly, the use of autophagy inhibitor CQ efficiently rescued mutp53 protein levels in sh/MKK3 cells (Figure 4e), strongly suggesting that autophagy, induced upon MKK3 depletion, may have major roles in mutp53 protein reduction.

### MKK3 depletion combined with chemotherapy decreases cell survival fractions and allows reducing dose in both wtp53 and mutp53 cancer cells

**Figure 5:**
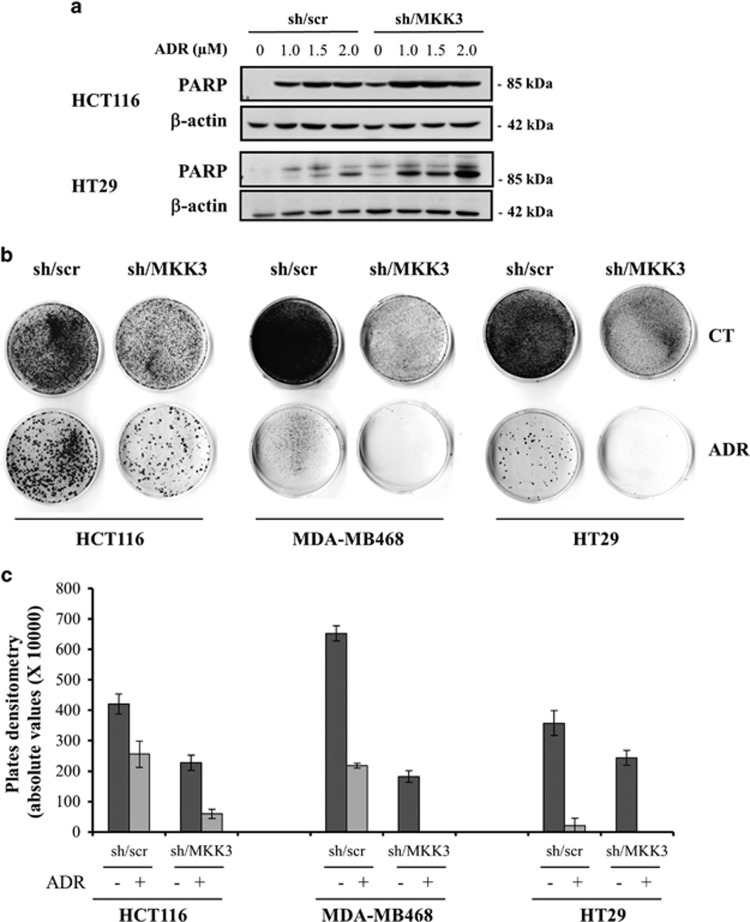
MKK3 depletion increases chemotherapeutic response in both wtp53 and mutp53 cancer cells. (**a**) Engineered sh/scr and sh/MKK3 HCT116 and HT29 cell lines were maintained in DOX condition for 48 h and treated/untreated for 24 h with ADR (1, 1.5, and 2 *μ*M). Cells were then collected and protein lysates were analyzed by western blot analysis. The membrane was probed with specific anti-PARP and anti-*β*-actin (as loading control) antibodies. **(b)** Clonogenic survival assay of: HCT116 (left panel), MDA-MB468 (middle panel), and HT29 (right panel) sh/scr and sh/MKK3 cell lines, DOX-induced for 48 h and then treated with ADR 0.1 *μ*M (HCT116 and MDA-MB468) or 0.5 *μ*M (HT29) for 24 h. After treatment, the culture medium was replenished, and cells were maintained at 37 °C for 14 days. Grown colonies were stained with crystal violet. (**c**) Densitometric analyses of clonogenic assays as described in **b**. Plates were scanned and quantified by the ImageJ software. The graph shows the absolute values of plates densitometry. Plating was performed in triplicate

Based on the achieved results, we investigated whether targeting MKK3 in combination with chemotherapy could improve therapeutic response. To this aim, the apoptotic response to different adriamycin (ADR) doses was analyzed in both wtp53 and mutp53 sh/MKK3 and sh/scr cells. As shown in Figure 5a, combined MKK3 knockdown with ADR treatment induced significantly higher poly (ADP-ribose) polymerase (PARP) cleavage in both wtp53 and mutp53 cells, with respect to ADR-treated control cells (sh/scr). Noteworthy, the lower dose of ADR in MKK3-depleted cells induces a significantly higher PARP cleavage with respect to control sh/scr cells challenged with the higher ADR dose with both wt and mutp53 cells (Figure 5a). Results are suggesting that MKK3 targeting combined to ADR treatment would provide a better therapeutic response allowing chemotherapeutic dose reduction in both wt and mutp53 cancer lines.

We next assessed long-term responses to ADR treatment by clonogenic assay. As shown in Figure 5b, the reduced clonogenic cell survival upon MKK3 depletion was markedly improved after ADR treatment, compared with ADR-treated control cells (sh/scr; Figure 5b), as also evidenced by densitometric analyses of clonogenic assays (Figure 5c). These findings reveal a potential additive effect of MKK3 depletion on tumor cell response to drugs, thus pointing at MKK3 as a novel potential clinical target to improve both wtp53 and mutp53 cancer cell response to chemotherapeutic agents.

### MKK3 depletion affects xenograft tumor growth and potentiates chemotherapeutic effect *in vivo*

**Figure 6:**
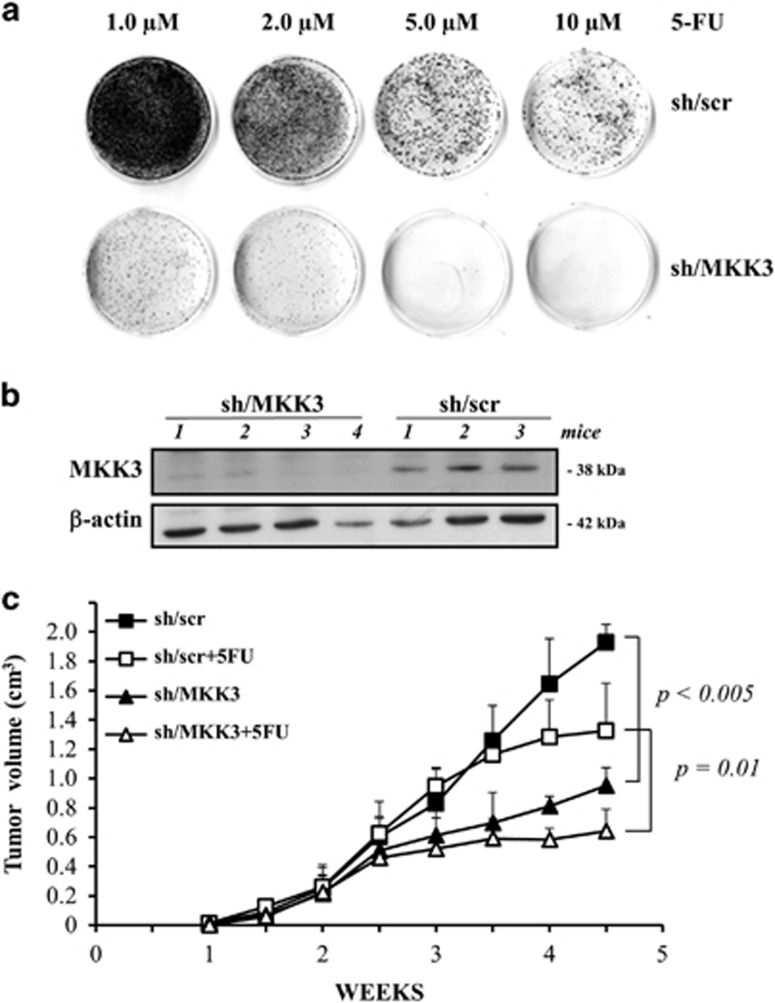
MKK3 depletion affects xenograft tumor growth and increases chemotherapeutic response *in vivo*. (**a**) Dose-response effect of 5-FU on clonogenic survival in HT29-sh/scr and sh/MKK3 cell lines, DOXI-induced for 48 h, and then treated with 5-FU (1, 2, 5, and 10 *μ*M) for 24 h. After treatment, the culture medium was replenished, and cells were maintained at 37 °C for 14 days. Grown colonies were stained with crystal violet. (**b**) Efficient MKK3 depletion was achieved *in vivo*, assessed by western blot analysis performed on a representative number of xenograft tumors generated with sh/scr and sh/MKK3 engineered HT29 cancer cells injected in nude mice (CD1/SWISS). Numbers identify single animal. (**c**) After tumor nodule formation, DOX (2.0 g/l, tap water) was delivered to all mice. To assess chemotherapeutic response in MKK3-depleted tumors, 5-FU (50 mg/kg, intraperitoneal) was delivered to a subgroup of sh/scr and sh/MKK3 tumor-bearing mice (8 mice/group). Tumor growth was followed by calliper measurements twice a week. Representative data of two independent experiments are reported. Student's *t*-test analyses were performed to assess significance between sh/scr and sh/MKK3 tumor-bearing mice (*P*<0.05) and along with 5-FU treatments (*P*=0.01)

To evaluate the MKK3 role in more physiological experimental models, we explored *in vitro* and *in vivo* HT29 cancer cell response to 5-fluorouracil (5-FU), a commonly used chemotherapeutic drug in colon cancer patients.^33^ In accordance to the above data with ADR, HT29 clonogenic cell survival was markedly and dose-dependently reduced after 5-FU treatment, compared with drug-treated control cells (sh/scr; Figure 6a). Noteworthy, control sh/scr cells challenged with higher 5-FU dose (10 *μ*M) generated a number of colonies significantly higher with respect to MKK3-depleted cells treated with the lower dose (1.0 *μ*M), further confirming the evidence that combined treatments allow chemotherapeutic dose reduction. Next, we generated tumor xenografts with HT29-sh/scr or -sh/MKK3 cells injected in nude mice, where MKK3 was efficiently reduced after DOX delivery (Figure 6b). *In vivo* results of tumor growth showed that MKK3 depletion *per se* significantly reduced tumor volume, compared with xenografts derived from control cells (sh/scr; Figure 6c, *P*<0.05); interestingly, MKK3 depletion further increased the effect of 5-FU on tumor growth (Figure 6c, *P*=0.01), in agreement with our hypothesis that MKK3 targeting could constitute a novel therapeutic strategy to improve tumor response to therapies.

## Discussion

MKK3 is a dual specificity protein kinase that belongs to the MAP kinase kinase family. This kinase is activated by mitogenic or stress-inducing stimuli and participates in the MAP kinase-mediated signaling cascade, leading to cell proliferation and survival. Recent findings suggested important roles for MKK3 in tumor invasion and progression. Accordingly, we previously identified MKK3 as a novel mutp53 target gene involved in tumor growth and survival in breast and colon cancer lines.^9^ In the present study, we wanted to evaluate whether MKK3 could play a role also in wtp53-bearing cells. We found that MKK3 depletion strongly inhibited proliferation and survival of wtp53-bearing cancer cell lines, whereas it did not have any relevant effects on untransformed cells. In the attempt to identify the molecular mechanisms involved in such biological outcomes, we found that MKK3 depletion affected several pathways: (i) it triggered ER stress and autophagy, (ii) stabilized wtp53, and (iii) degraded mutp53.

Autophagy is a degradative process through which damaged organelles and misfolded proteins are targeted for disruption via the lysosomes. In cancer, autophagy may contribute to tumor cell survival. In established tumors, autophagy might act as a pro-survival pathway in response to metabolic stresses such as nutrient deprivation, hypoxia, absence of growth factors, and in the presence of chemotherapy or some targeted therapies that might mediate resistance to anticancer therapies.^34, 35, 36^ However, persistent or excessive autophagy is also shown to promote cell death following treatments with specific chemotherapeutic agents or radiotherapy, either by enhancing the induction of apoptosis or mediating ‘autophagic cell death'.^37^ In agreement, we found here that MKK3 depletion induced autophagic cell death, as assessed by LC3 stabilization and p62 degradation, and that blocking autophagy either with CQ or ATG5 silencing significantly reduced the cell death upon MKK3 depletion. This is also in accord with previously reported data showing the detrimental effect of MAPK14/p38a inhibition on proliferation and survival of colorectal cancer cells, leading to cell cycle arrest and autophagy-mediated cell death.^10^

Successful cancer eradication often needs the combination of different anticancer strategies to overcome chemoresistance and/or improve chemosensitivity. To explore whether MKK3 depletion might impact on tumor cell response to anticancer drugs, we demonstrated that MKK3 knockdown improves response to therapies, in both wtp53 and mutp53 cancer cells, allowing chemotherapeutic dose reduction.

Interestingly, we found that MKK3 deficiency induced, in wtp53-bearing cancer cells, an evident stabilization of p53 protein level that was straightly linked to ER stress induction, according to recent data.^38^ However, p53 stabilization does not contribute in p21 gene expression and authophagy as its depletion does not impact on the biological effects observed upon MKK3 depletion (Figures 2d and e and data not shown).

In conclusion, our study demonstrates that MKK3 targeting has an important effect in reducing tumor cells proliferation and survival, both *in vitro* and *in vivo*, without affecting normal cells. Noteworthy, MKK3 depletion showed a potential additive effect with chemotherapeutic drugs on reducing tumor growth, likely through different triggered mechanisms. These data suggest the potential use of MKK3 inhibitors as an adjuvant therapy to potentiate the efficiency of chemotherapies in non-responder patients, although further studies will be necessary to confirm our hypothesis.

## Materials and Methods

### Cell lines

The human lines HCT116 (colorectal carcinoma),^39^ FB1329 (human fibroblast),^40^ H1299 (non-small-cell lung carcinoma),^9^ MDA-MB468 (breast adenocarcinoma),^9^ and engineered HT29 (colon adenocarcinoma)-sh/scr and -sh/MKK3 sublines^9^ were cultured in Dulbecco's modified Eagle's medium (Eurobio, Les Ulis, France), whereas the MCF7 line (breast adenocarcinoma)^41^ was cultured in Dulbecco's modified Eagle's medium-F12 (1 : 1). All tissue culture media were supplemented with 10% fetal bovine serum (GIBCO-BRL, Grand Island, NY, USA), L-glutamine (2 mM), and Penicillin/Streptomycin (100 U/ml; Life Technologies Inc., Eggenstein, Germany). The MCF10A line (normal breast epithelial; kindly provided from Dr S Anastasi) was cultured in MEGM supplemented with bovine pituitary extract (52 *μ*g/ml), hydrocortisone (0.5 *μ*g/ml), hEGF (10 ng/ml), and insulin (5 *μ*g/ml; MEGM Bullet Kit, Lonza Corporation, Walkersville, MD, USA). All lines were grown at 37 °C in a humidified atmosphere with 5% CO_2_.

### Lentiviral infection

HCT116, MCF7, MDA-MB468, H1299, FB1329, and MCF10A lines have been engineered with a lentiviral-based TET-OFF inducible RNA interference system carrying shRNA sequences specific to human MKK3 (sh/MKK3) or control scrambled (sh-scr) as previously described.^9^ To induce shRNA expression, all engineered -sh/scr and -sh/MKK3 sublines were challenged with DOX (Sigma-Aldrich, St. Louis, MO, USA; 1 *μ*g/ml), added to the culture medium after seeding and freshly added every 3 days.

### Transfections

siRNAs specific for human ATG5 as well as siRNA unrelated to the human genome were purchased from Dharmacon (Thermo Scientific, Milan, Italy) and delivered to cells as elsewhere described.^27^

To deplete endogenous wtp53 in HCT116-sh/MKK3 sublines, cells were transfected with pRetroSuper vector carrying sh/RNA specific to p53 (sh/p53)^42^ or control (sh/RNA). Transfections were performed with Lipofectamine/Plus reagent (Invitrogen, Monza, Italy) following manufacture's instruction. Then, 48 h post transfection, cells were selected with Puromycin (2 *μ*g/ml; Sigma-Aldrich) to generate mixed population.

### Cell proliferation and survival analyses

Cells were seeded (2.0 × 10^4^/6-well plates) and induced with DOX (1 *μ*g/ml). After 48 h, in a time-course-dependent manner, both floating and adherent cells were collected, stained with 0.4% Trypan blue reagent (Sigma, St. Louis, MO, USA), and counted to determine cell proliferation and viability with hemocytometer. All the experiments were performed in triplicate.

To determine the percentage of apoptotic cell death along with ATG5 depletion, 25 *μ*l of cell suspensions were mixed with 1 *μ*l of dye mix (DAPI, 1 *μ*g/ml+ethidium bromide, 100 *μ*g/ml). The mixture was placed on a microscope slide, covered with a 22-mm^2^ coverslip and slides examined with ‘Zeiss Axioskop 2 plus' fluorescent microscope. For each sample, 200 cells were counted and recorded as V (viable cells), NVN (non-viable cells with normal nuclei), and NVA (non-viable cells with apoptotic nuclei) characterized by highly condensed or fragmented nuclei. The % of apoptotic dead cells was then calculated as follow: % apoptotic cell=100 × NVA/(VA+NVN+NVA).

### Semi-quantitative reverse transcriptase–PCR

Cells were seeded (1.5 × 10^5^/60 mm dish) and induced with DOX (1 *μ*g/ml) for 72 h, unless otherwise indicated. Total RNAs, extracted with TRIzol (15596-026; Invitrogen), were retro-transcribed with Moloney-Murine-Leukemia virus reverse transcriptase (M-MLV-RT, Invitrogen) following the manufacturer's instruction. For semi-quantitative PCR, cDNAs were amplified by Hot-Master Taq (5PRIME) with specific set of primers: *hGAPDH* (FOR5′-*ATGACATCAAGAACGTGGTG*-3′, REV5′-*CATACCAGGAAATGAGCTTG*-3′); *hMKK3* (FOR5′- *GTGGAGCCCGCAGTCCTCTA*-3′, REV5′-*GGGTGGCTTGGACATGCAG*-3′); *hp21* (FOR5′-*CCCCTTCGGCCCGGTGGAC*-3′, REV5′-*CCGTTTTCGACCCTGAGAG*-3′); *hp53* (FOR5′-*GTCTGGGCTTCTTGCATTCT*-3′, REV5′-*AATCAACCCACAGCTGCAC*-3′); and *hCHOP* (FOR5′- *GCACCTCCCAGAGCCCTCACTCTCC*-3′, RE5′-*GTCTACTCCAAGCCTTCCCCCTGCG*-3′).

### Western blotting

Cells (1.5 × 10^5^ cells/60 mm dishes) were washed twice in ice-cold PBS, harvested by scraping, and then lysed in 1 × RIPA buffer (150 mm NaCl, 1% Triton X-100, 0.25% sodium deoxycholate, 0.1% SDS, 50 mm Tris/HCl, pH 8.0, and 20 mm EDTA) supplemented with 1 × protease and phosphatase inhibitor mixture (Sigma-Aldrich), 1 mm phenylmethylsulfonyl fluoride (Sigma-Aldrich), 50 mm sodium fluoride (Sigma), and 50 mm dithiothreitol (Bio-Rad, Hercules, CA, USA). Lysates were incubated for 30 min in ice, clarified by centrifugation, and resolved onto 10 or 18% SDS–polyacrylamide gel electrophoresis; 30 *μ*g/lane). Blotting was performed according to the standard protocols, and filters were immuno-reacted with the following antibodies: rabbit monoclonal anti-MKK3 (D4C3; 1 : 1000; Cell Signaling), mouse anti-p53 (DOI),^43^ rabbit polyclonal anti-Phospho-p53 (Ser392; 1 : 1000; Ser51; Cell Signaling), mouse anti-actin (Ab-1; Calbiochem, San Diego, CA, USA), and rabbit anti-p21 (Santa Cruz Biotechnology, Dallas, TX, USA); rabbit polyclonal anti-LC3 (1 : 1000; Sigma-Aldrich), mouse monoclonal anti-p62 (SQSTM1; 1 : 1000; Santa Cruz Biotechnology, Dallas, TX, USA), rabbit monoclonal anti-GRP78/BiP (C50B12; 1 : 1000; Cell Signaling), rabbit monoclonal anti-IRE1*α* (14C10; 1 : 1000; Cell Signaling), rabbit polyclonal anti-Phospho-EIF2*α* (pEIF2*α*; 1 : 1000; Ser51; Cell Signaling), rabbit polyclonal anti-EIF2*α* (1 : 1000; Cell Signaling), rabbit polyclonal anti-cleaved Caspase-3 (Asp175; 1 : 1000; Cell Signaling), rabbit polyclonal anti-ATG5 (1 : 1000; Sigma-Aldrich), and rabbit monoclonal anti-PARP (Poly-ADP-ribose polymerase) p89 Fragment (Asp214; 1 : 1000; Cell Signaling). Secondary HRP-conjugated anti-mouse or anti-rabbit (Bio-Rad) antibodies were used. Detection of immuno-reactions was performed by ECL kit (Amersham Biosciences, Glattbrugg, Switzerland). Images were acquired with the EPSON Expression 10000 XL scanner (Epson, Long Beach, CA, USA) and densitometry was performed with the ImageJ software (NIH, Bethesda, MD, USA).

### Chemical autophagy inhibition

MCF7-sh/scr and -sh/MKK3 sublines were seeded (1.5 × 10^5^ cells/60 mm dishes) and supplemented with DOX. Then, 24 h later, cells were treated/untreated with CQ (25 *μ*M, Sigma-Aldrich), and collected subsequently at 96 h of total DOX induction for cells viability analyses. To inhibit autophagy in MDA-MB468-sh/scr and -sh/MKK3 sublines, cells were plated (1.5 × 10^5^ cells/60 mm dishes) and supplemented with DOX. Seventy-two hours later cells were treated/untreated with CQ (25 *μ*M), and collected for western blot analyses 48 h later.

### Chemotherapeutic treatments

To study apoptotic response, HCT116- and HT29-sh/scr and sh/MKK3 sublines were plated (1.5 × 10^5^ cells/60 mm dishes) and supplemented with DOX. Fourth-eight hours later, cells were challenged with Adriamycin/Doxorubicin (ADR; Pharmacia, Milan, Italy) and collected 24 h later for western blot analyses.

For clonogenic survival assay, engineered -sh/scr and -sh/MKK3 sublines were seeded along with DOX as follow: HCT116 (4.0 × 10^4^ cells/60 mm dishes), MDA-MB468, and HT29 (1.5 × 10^5^ cells/60 mm dishes). Fourthly-eight hours later, cells were challenged with either ADR (0.1- 0.5 *μ*M) or 5-FU (1, 2, 5, and 10 *μ*M; Roche, Milan, Italy). Then, 24 h later, cells were washed three times with PBS and fed with complete medium supplemented with DOX, which was replenished every 72 h. Fourteen days later, colonies were stained with crystal violet and analyzed by densitometry with ImageJ software. Experiments were performed in triplicate and repeated three times.

### *In vivo* assay

Exponentially growing HT29-sh/scr and sh/MKK3 sublines were injected (5 × 10^5^ cell/mouse) subcutaneously in 45-day-old female nude mice (CD1/Swiss, Charles River, Lecco, Italy). Two weeks later, which tumors reached a volume of 0.2 cm^3^, all mice were delivered with DOX (2.0 g/l) as reported.^43^ Animals bearing HT29-sh/scr or sh/MKK3 tumors were randomly subdivided into groups (8 mice/group), and either treated or untreated with 5-FU (50 mg/kg) by intraperitoneal injection at days 7, 9, 11 after DOX delivering started. Tumor growth was followed by caliper measurements twice a week and tumor volumes (TV) estimated by the formula: TV=*a* × (*b*^2^)/2, where *a* and *b* are tumor length and width, respectively. At the end of the experiment, all the animals were killed, tumors excised and analyses by western blot analysis to ascertain the occurred MKK3 depletion *in vivo*. All the procedures involving animals and their care were approved by the Ethical Committee of the Regina Elena Cancer Institute (CE/532/12) and were conformed to the relevant regulatory standards in accordance with the Italian legislation.

### Statistical analysis

All experiments were performed in triplicate. Numerical data are reported as means±S.D.s. Student's *t*-test was used for statistical significance of the differences between treatment groups. Statistical analysis was performed using analysis of variance at 5% (*P*<0.05) or 1% (*P*<0.01).

## Abbreviations

MAP2K3: MKK3, mitogen-activated protein kinase kinase 3
Mut: mutant
Wt: wild type
ER: endoplasmic reticulum
PARP: poly (ADP-ribose) polymerase
PERK/eIF2*α*: PKR-like ER kinase/eukaryotic translation initiation factor 2*α*
IRE1: Inositol-requiring Enzyme 1
LC3: microtubule-associated protein 1 light chain 3
TET: tetracycline
DOX: doxycycline
sh/scr: short hairpin/scramble
CQ: Chloroquine
CHOP: CCAAT-enhancer-binding protein homologous protein
GRP78/Bip: glucose-regulated protein 78/immunoglobulin heavy chain-binding protein
ATG5: autophagy protein 5
ADR: adriamycin
5-FU: 5-fluorouracil
MAPK: mitogen-activated protein kinase
